# Supplementary material for: Cultural adaptation, translation and validation of the conflict in adolescence dating relationship inventory (CADRI) in the Greek language
Source: PLoS One. 2025 Feb 4;20(2):e0317833. doi: 10.1371/journal.pone.0317833 (PMC11793791; doi:10.1371/journal.pone.0317833)
Supplement: S1 File — (PDF) [file pone.0317833.s001.pdf]

CONFLICT IN ADOLESCENT DATING RELATIONSHIPS (CADRI) GREEK VERSION

Η καταγραφή των συγκρούσεων στις εφηβικές συντροφικές σχέσεις

Εισαγωγικές ερωτήσεις γνωριμιών

Παρακαλείσθε να επιλέξετε την απάντηση που ισχύει καλύτερα για εσάς.

- Δεν έχω αρχίσει ακόμα να βγαίνω σε ραντεβού.
- Έχω αρχίσει να βγαίνω και είχα αγόρι/ή κορίτσι.

Παρακαλείσθε να επιλέξετε όλα τα παρακάτω πλαίσια που περιγράφουν τα είδη των σχέσεων/γνωριμιών που βιώνετε αυτήν τη στιγμή και εκείνων που έχετε βιώσει κατά το παρελθόν:

- έξοδοι με παρέες ανδρών/γυναικών
- ραντεβού με διαφορετικούς ανθρώπους
- ραντεβού με ένα άτομο χωρίς συγκεκριμένη δέσμευση
- ραντεβού αποκλειστικά με ένα άτομο
- αρραβωνιασμένος

Αν είχατε ποτέ σχέση ή βγαίνατε με κάποιον κατά το στάδιο της γνωριμίας, παρακαλείσθε να απαντήσετε στις ακόλουθες ερωτήσεις:

Σε ποια ηλικία ξεκίνησες να βγαίνεις/ή να έχεις αγόρι/κορίτσι; ____________________

Πόσα αγόρια/ ή κορίτσια είχατε; (χωρίς να συμπεριλαμβάνονται τα παιδικά ερεθίσματα_

Πόσους φίλους είχες/έχεις κατά τη διάρκεια:

Β Γυμνασίου εβδομάδων/ή μηνών εβδομάδων/ή μηνών

Αριθμός αγοριών/ή κοριτσιών ____ Η μεγαλύτερη σχέση ___________Η πιο σύντομη σχέση __________

Γ Γυμνασίου

Αριθμός αγοριών/ή κοριτσιών ____ Η μεγαλύτερη σχέση ___________Η πιο σύντομη σχέση __________

Α Λυκείου

Αριθμός αγοριών/ή κοριτσιών ____ Η μεγαλύτερη σχέση ___________Η πιο σύντομη σχέση __________

Β/Γ Λυκείου

Αριθμός αγοριών/ή κοριτσιών ____ Η μεγαλύτερη σχέση ___________Η πιο σύντομη σχέση __________

Οι επόμενες σελίδες σας ζητούν να απαντήσετε σε ερωτήσεις σχετικά με τον τωρινό ή τον/την πρόσφατο/η πρώην φίλο/-η σας. Παρακαλείσθε να εστιάσετε σε συγκεκριμένο άτομο το οποίο θα σκέφτεστε όταν απαντάτε σε αυτές τις ερωτήσεις.

- Σκέφτομαι κάποιον/α που είναι φίλος/η μου αυτή τη στιγμή. (Παρακαλείσθε να μεταβείτε στο Α)
- Σκέφτομαι έναν/μία πρόσφατο/η πρώην φίλο/η (σε χρονικό διάστημα έως τους τελευταίους 3 μήνες). (Παρακαλείσθε να μεταβείτε στο Β, επόμενη σελίδα)
- Σκέφτομαι έναν/ή μία πρώην φίλο/η από τον περασμένο χρόνο. (Παρακαλείσθε να μεταβείτε στο Β).

Α) Αν αυτός/ή είναι ο/η τωρινός/ή φίλος/η σας:

Πόσο καιρό βγαίνετε σε ραντεβού ή βγαίνετε ως φίλοι στο αρχικό στάδιο γνωριμίας; _____

Πόσο συχνά βλέπετε ο ένας τον άλλον; *Παρακαλείσθε να κυκλώστε την καλύτερη απάντηση.*

-Κάθε μέρα στο σχολείο
-Κάθε μέρα στο σχολείο και κάθε μέρα εκτός σχολείου
-2-3 φορές την εβδομάδα
-Μία φορά την εβδομάδα ή λιγότερο

Πόσο χρόνο περνάτε μόνοι μαζί; _________ώρες την ημέρα ή ________ώρες την εβδομάδα

Τι είδους πράγματα κάνετε μαζί; ____

Πόσο συχνά μαλώνετε ή διαφωνείτε; ________φορές/ ημέρα ή __________φορές/εβδομάδα

Για ποια πράγματα μαλώνετε ή διαφωνείτε;_______________________________ ________________

-Πόσο χρονών είναι ο/η ίδιος/α ; ___________

Πόσο σημαντική είναι αυτή η σχέση για εσάς; (Παρακαλείσθε να κυκλώσετε μια από τις παρακάτω απαντήσεις).
Όχι πολύ σημαντικό -----Κάπως σημαντικό------ Σημαντικό -----Πολύ σημαντικό

Παρακαλείσθε να επιλέξετε μία από τις ακόλουθες πέντε κατηγορίες που περιγράφει καλύτερα τον/την σύντροφο τον/την οποίο/α σκέφτεστε όταν συμπληρώνετε αυτό το ερωτηματολόγιο:

- βγαίνοντας σε παρέες ανδρών/γυναικών
- ραντεβού με διαφορετικούς ανθρώπους
- ραντεβού με ένα άτομο χωρίς συγκεκριμένη δέσμευση
- ραντεβού αποκλειστικά με ένα άτομο
- αρραβωνιασμένος

B) Αν αυτός/-ή είναι ο/η πρώην φίλος/-η σου:

Πόσο καιρό βγαίνατε μαζί;_ __________
Πόσο συχνά βλέπατε ο ένας τον άλλον; *Παρακαλείσθε να κυκλώσετε την καλύτερη απάντηση παρακάτω.*
-Κάθε μέρα στο σχολείο
-Κάθε μέρα στο σχολείο και κάθε μέρα εκτός σχολείου
-2-3 φορές την εβδομάδα
-Μία φορά την εβδομάδα ή λιγότερο
Πόσο χρόνο περάσατε μόνοι μαζί; ________ώρες την ημέρα **Ή** _______ώρες την εβδομάδα.
Τι είδους πράγματα κάνατε μαζί; _______________
Πότε σταματήσατε να βγαίνετε μαζί/ ή να βλέπετε ο ένας τον άλλον; _______________
Γιατί σταματήσατε να βγαίνετε μαζί του/της; ___________________
Πόσο συχνά μαλώνατε/διαφωνούσατε μαζί του/της; _______φορές/ ημέρα ή _________φορές/εβδομάδα
Για ποια πράγματα μαλώνατε ή διαφωνούσατε; _____________
Πόσο χρονών ήταν αυτός/ή; _______
Πόσο σημαντική ήταν αυτή η σχέση για εσάς; *Παρακαλείσθε να κυκλώσετε μια από τις παρακάτω απαντήσεις*
Όχι πολύ σημαντικό -----Κάπως σημαντικό------ Σημαντικό -----Πολύ σημαντικό

Παρακαλείσθε να επιλέξετε μία από τις ακόλουθες πέντε κατηγορίες που περιγράφει καλύτερα τον/την σύντροφο που σκέφτεστε όταν συμπληρώνετε αυτό το ερωτηματολόγιο:

- βγαίνοντας σε παρέες ανδρών/γυναικών
- ραντεβού με διαφορετικούς ανθρώπους
- ραντεβού με ένα άτομο χωρίς συγκεκριμένη δέσμευση
- ραντεβού αποκλειστικά με ένα άτομο
- αρραβωνιασμένος/-η

<u>Η καταγραφή των συγκρούσεων στις εφηβικές συντροφικές σχέσεις</u>

Οι παρακάτω ερωτήσεις σας ζητούν να απαντήσετε για γεγονότα που μπορεί να σας συνέβησαν με το αγόρι/κορίτσι σας, ενώ τσακωνόσασταν μαζί του/της.
Παρακαλείσθε να επιλέξετε το πλαίσιο το οποίο αποδίδει την καλύτερη εκτίμηση για το πόσο συχνά συνέβησαν αυτά τα γεγονότα με τον/την νυν ή τον/την πρώην φίλο/η σας τον περασμένο χρόνο.
Είναι σημαντικό να θυμάστε ότι όλες οι απαντήσεις είναι εμπιστευτικές.

Ως οδηγός ερμηνείας της κλίμακας παρακαλείσθε να χρησιμοποιήσετε τα ακόλουθα στοιχεία:

- **Ποτέ:** αυτό δεν έχει συμβεί ποτέ στη σχέση σας
- **Σπάνια:** αυτό έχει συμβεί μόνο 1-2 φορές στη σχέση σας
- **Μερικές φορές:** αυτό έχει συμβεί περίπου 3-5 φορές στη σχέση σας
- **Συχνά:** αυτό έχει συμβεί 6 ή περισσότερες φορές στη σχέση σας

**Κατά τη διάρκεια μιας σύγκρουσης ή λογομαχίας με τον/την φίλο/η μου τον περασμένο χρόνο:**		**Ποτέ**	**Σπάνια**	**Μερικές φορές**	**Συχνά**
1.Α	Έδωσα λόγους από τη δική μου πλευρά για την έναρξη του καυγά.				
1.Β	Έδωσε λόγους από τη δική του/της πλευρά για την έναρξη του καυγά.				
2.Α	Τον/την άγγιξα σεξουαλικά όταν δεν ήθελε να το κάνω.				
2.Β	Με άγγιξε σεξουαλικά όταν δεν ήθελα να το κάνει.				
3.Α	Προσπάθησα να στρέψω τους φίλους του/της εναντίον του/της.				
3.Β	Προσπάθησε να στρέψει τους φίλους μου εναντίον μου.				
4.Α	Έκανα κάτι για να τον/την κάνω να νιώσει ζήλια.				
4.Β	Έκανε κάτι για να με κάνει να νιώσω ζήλια.				
5.Α	Κατέστρεψα ή απείλησα να καταστρέψω κάτι που είχε αξία για τον/την ίδιο/α.				
5.Β	Κατέστρεψε ή απείλησε να καταστρέψει κάτι που είχε αξία για εμένα.				
6.Α	Του/της είπα ότι φταίω εν μέρει.				
6.Β	Μου είπε ότι έφταιξε εν μέρει.				
7.Α	Ανέφερα κάτι κακό που είχε κάνει στο παρελθόν.				
7.Β	Ανέφερε κάτι κακό που είχα κάνει στο παρελθόν.				
8.Α	Του/της πέταξα κάτι (ένα αντικείμενο).				
8.Β	Μου πέταξε κάτι (ένα αντικείμενο).				
9.Α	Είπα πράγματα με σκοπό να τον/την θυμώσω.				
9.Β	Είπε πράγματα με σκοπό να με θυμώσει.				
10.Α	Ανέφερα τις αιτίες για τις οποίες πίστευα ότι έκανε λάθος.				
10.Β	Ανέφερε τις αιτίες για τις οποίες πίστευε ότι έκανα λάθος.				
11.Α	Συμφώνησα ότι είχε εν μέρει δίκιο.				
11.Β	Συμφώνησε ότι είχα εν μέρει δίκιο.				
12.Α	Του/της μίλησα με εχθρικό ή κακόβουλο τόνο φωνής.				
12.Β	Μου μίλησε με εχθρικό ή κακόβουλο τόνο φωνής.				
13.Α	Τον/την ανάγκασα να κάνει σεξ όταν δεν ήθελε να κάνει.				
13.Β	Με ανάγκασε να κάνω σεξ όταν δεν ήθελα να κάνω.				
14.Α	Πρόσφερα μια λύση που πίστευα ότι θα μας έκανε και τους δύο χαρούμενους.				

14.Β	Πρόσφερε μια λύση που πίστευε ότι θα μας έκανε και τους δύο χαρούμενους.				
15.Α	Τον/την απείλησα σε μια προσπάθεια να κάνω σεξ μαζί του/της.				
15.Β	Με απείλησε σε μια προσπάθεια να κάνει σεξ μαζί μου.				
16.Α	Σταμάτησα να μιλάω μέχρι να ηρεμήσουμε.				
16.Β	Σταμάτησε να μιλάει μέχρι να ηρεμήσουμε.				
17.Α	Τον/την πρόσβαλα με υποτιμητικές εκφράσεις ή λέξεις.				
17.Β	Με πρόσβαλε με υποτιμητικές εκφράσεις ή λέξεις.				
18.Α	Συζήτησα το θέμα με ήρεμο τρόπο.				
18.Β	Συζήτησε το θέμα με ήρεμο τρόπο.				
19.Α	Τον/την φίλησα όταν δεν ήθελε να το κάνω.				
19.Β	Με φίλησε όταν δεν ήθελα να το κάνει.				
20.Α	Είπα πράγματα στους φίλους του/της για αυτόν/ην προκειμένου να τους στρέψω εναντίον του/της.				
20.Β	Είπε πράγματα στους φίλους μου για εμένα προκειμένου να τους στρέψει εναντίον μου.				
21.Α	Τον/την κορόιδευα ή τον διακωμωδούσα μπροστά σε άλλους.				
21.Β	Με κορόιδευε ή με διακωμωδούσε μπροστά σε άλλους.				
22.Α	Του/της ανέφερα πόσο στενοχωρημένη/ος ήμουν.				
22.Β	Μου ανέφερε πόσο στενοχωρημένος ήταν.				
23.Α	Παρακολούθησα με ποιους ήταν και πού ήταν.				
23.Β	Παρακολουθούσε με ποιους ήμουν και πού ήμουν.				
24.Α	Τον/την κατηγόρησα για το πρόβλημα.				
24.Β	Με κατηγόρησε για το πρόβλημα.				
25.Α	Τον/την κλώτσησα, τον/την χτύπησα ή τον/την γρονθοκόπησα.				
25.Β	Με κλώτσησε, με χτύπησε ή με γρονθοκόπησε.				
26.Α	Έφυγα από τον χώρο για να ηρεμήσω.				
26.Β	Έφυγε από τον χώρο για να ηρεμήσει.				
27.Α	Υποχώρησα προκειμένου να αποφύγω τη σύγκρουση.				
27.Β	Υποχώρησε προκειμένου να αποφύγει τη σύγκρουση				
28.Α	Τον/την κατηγόρησα ότι φλέρταρε με άλλη/ο κοπέλα/αγόρι.				
28.Β	Με κατηγόρησε ότι φλέρταρα με άλλον/η αγόρι/κοπέλα.				
29.Α	Προσπάθησα σκοπίμως να τον/την τρομάξω.				
29.Β	Προσπάθησε σκοπίμως να με τρομάξει.				
30.Α	Τον/την χαστούκισα ή του/της τράβηξα τα μαλλιά.				
30.Β	Με χαστούκισε ή μου τράβηξε τα μαλλιά.				
31.Α	Απείλησα να τον/την πληγώσω.				
31.Β	Απείλησε να με πληγώσει.				
32.Α	Απείλησα να τερματίσω τη σχέση.				
32.Β	Απείλησε να τερματίσει τη σχέση.				
33.Α	Απείλησα ότι θα τον/την χτυπήσω ή θα του/της πετάξω κάποιο αντικείμενο.				
33.Β	Απείλησε να με χτυπήσει ή να μου πετάξει κάποιο αντικείμενο.				
34.Α	Τον/την έσπρωξα ή τον/την ταρακούνησα.				
34.Β	Με έσπρωξε ή με ταρακούνησε.				
35.Α	Διέδωσα φήμες για αυτόν/ή.				
35.Β	Διέδωσε φήμες για μένα.				
